# Dehydroandrographolide enhances innate immunity of intestinal tract through up-regulation the expression of hBD-2

**DOI:** 10.1186/s40199-015-0119-4

**Published:** 2015-07-30

**Authors:** Wen-Bi Xiong, Zhen-Jun Shao, Yao Xiong, Jian Chen, Yun Sun, Ling Zhu, Li-Ming Zhou

**Affiliations:** Department of Pharmacology, West China Medical Center, Preclinical and Forensic Medical College, Sichuan University, Chengdu, 610041 P.R. China

**Keywords:** Innate immunity, Dehydroandrographolide, hBD-2, Antimicrobial peptide, defensin, p38 MAPK

## Abstract

**Background:**

Dehydroandrographolide (DA) is one of major active components in the well-known oriental herbal medicine *Andrographis paniculata* (Burm.f) Nees which belongs to the Acanthaceae family. DA is used for the treatment of infections in China. However, DA has not been found to significantly inhibit bacterial and viral growth directly. The current study investigates the effect of DA on the expression of human β –defensin-2 (hBD-2) in human intestinal epithelial cells and the possible signaling pathways.

**Methods:**

Human intestinal epithelial HCT-116 cells were incubated with 1–100 μM DA for 2–24 h. RT–PCR and Western blot were used to assess the expression of hBD-2. The specific inhibitors were used and the levels of phosphorylation of signaling molecules were detected for dissecting the signaling pathways leading to the induction of hBD-2.

**Results:**

MTT assay showed there was no obvious cytotoxicity for HCT-116 cells by 1–100 μM DA treatment. RT-PCR and Western blot assays showed that DA (1–100 μM) could up-regulate the expression of hBD-2, and the effect lasted longer than 24 h. By using SB203580 and SB202190 (inhibitors of p38), the enhancement of hBD-2 expression were significantly attenuated. However, inhibitor of ERK and inhibitor of JNK could not block the effect of DA. Furthermore, Western blot found activation of p38 but not ERK and JNK in DA-treated HCT-116 cells.

**Conclusion:**

The results suggested that DA enhanced innate immunity of intestinal tract by up-regulating the expression of hBD-2 through the p38 MAPK pathways.

## Background

*Andrographis paniculata* (Burm. f) Nees, which belongs to the Acanthaceae family, is an oriental herbal medicine that has been widely used in China, India, and other Southeastern Asian countries for hundreds of years due to its significant antipyretic and anti-inflammatory effect. In China, *A. paniculata* is often used for treatment of chronic or repeated onset infection and inflammation, especially for the upper respiratory tract infection and gut diarrhea [1–3] . Both andrographolide (Androg) and dehydroandrographolide (DA) are main active components in *A. paniculata*, which have been demonstrated to contribute anti-infectious property of the *A. paniculata* . Contemporary laboratory tests have exhibited their anti-inflammatory, antipyretic, antimicrobial, antiviral and immunostimulant capability [4–6]. In China, Androg and DA are called “Natural Antibiotics” [7]. However, the mechanism has not been fully clarified and Androg and DA have not been found to significantly inhibit bacterium and virus growth directly [8]. Our recent work demonstrated that DA could enhance innate immune function by up-regulating the expression of human β –defensin (hBD-1) of human lung epithelial cells [9].

Innate immunity plays a very important role for mucous membranes in defense against invading pathogenic microbes. The human gut harbors trillions of microbes which are essential for mediating physiology, metabolism and host immune responses. In normal condition, the microbes could coexist peacefully with the body owing to the innate immunity despite the intestinal mucosal surface acting as a primary barrier [10]. Hence, it is a good choice to enhance intestinal innate immune function for treatment of intestinal infection rather than using antibiotic for less antibiotic resistance and dysbacteriosis, and it is an alternative strategy to look for active ingredients to enhance the body's innate immunity from natural products such as ellagic acid [11], paeoniflorin [12], avocado sugar [13] etc.

Defensins, one of the 3 major antimicrobial peptides (AMPs) families, are important moleculars in innate immune system. Defensins are small (Mr 2 000 ~ 6 000 Da), cationic peptides containing disulfide bonds, so can interact with the membrane of invading microbes and active against many Gram-negative and Gram-positive bacteria, fungi, and enveloped viruses [14]. According to the position of their disulfide bonds human defensins are further classified as α- and β-defensins. Human β-Defensin (hBD) has six members which are expressed by many types of epithelial cells, including enterocytes. hBD-1, firstly discovered in 1995, is an important antimicrobial peptide against infection in human prostate, kidney and urogenital tract luminal epithelium [15]. hBD-2, discovered in 1997 [16], is the first discovered inducible human antimicrobial protein. Moreover, it was shown that hBD-2 had a broad spectrum of antimicrobial activity *in vitro* [17]. Therefore, in this study, we investigated the role of DA in hBD-2 expression in HCT-116 intestinal epithelial cells and the possible mechanism.

## Materials and methods

### Specific reagents

**Fig. 1:**
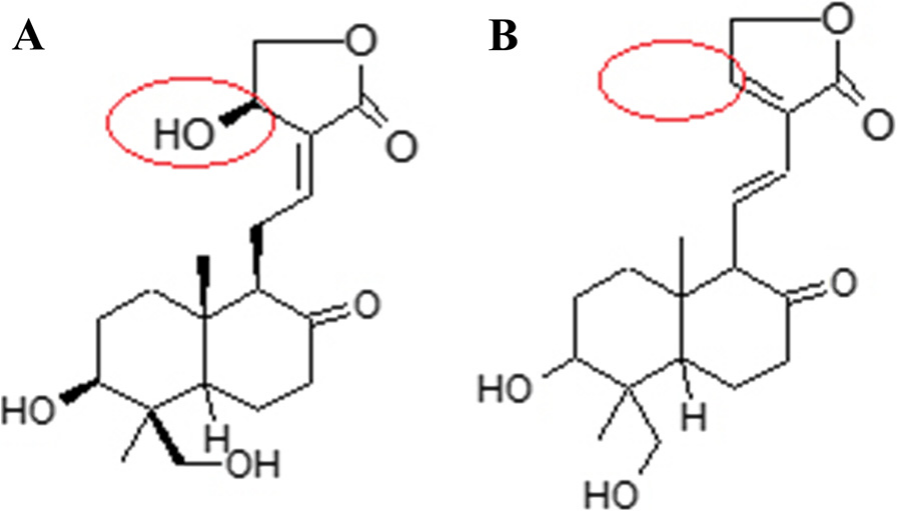
Molecular structure of Androg (**a**) and DA (**b**)

DA (Fig. 1) was obtained from the Sichuan Academy of Chinese Medical Sciences. The purity was 99.97 % as determined by high-performance liquid chromatography. DA was dissolved in dimethylsulphoxide (DMSO) at a concentration of 200 mmol/L. The final concentration of DMSO in the medium was not more than 0.1 % (v/v). The inhibitors PD98059 (an inhibitor of ERK), SB203580 and SB202190 (two different inhibitors of p38), SP600125 (an inhibitor of JNK) were purchased from Beyotime (Jiangsu, China). SB203580 is a pyridinyl imidazole derivative that specifically suppresses phosphorylation of p38. SB202190 is a highly potent and cell-permeable inhibitor of p38 that selectively inhibits the p38 a and b isoforms. Each inhibitor was reconstituted in DMSO and added to the culture medium so that the final concentration of DMSO was less than 0.1 % of the total volume.

### Cell line and culture

HCT-116 intestinal cells, a human cell line derived from the colonic epithelial cell line, were maintained at 37 °C in a humidified atmosphere of 95 % air and 5 % CO_2_ in RPMI 1640 medium (Gibco-BRL, Grand Island, NY). The medium mixture contains 10 % fetal bovine serum, 100 U/ml penicillin G and 100 μg/ml streptomycin. After reaching confluence, cells were seeded onto six well plates and incubated with DA. For all experiments, DA were added to cells on ice and incubated for 10 min, allowing DA to settle onto the cells, and then incubated at 37 °C in 5 % CO_2_ for the specified intervals. Cells treated with lipopolysaccharide (LPS, Sigma-Aldrich, St. Louis, Mo., USA) were employed as positive control. Vehicle control with equal concentration of DMSO was always included. HCT-116 intestinal cells were exposed to the specified concentrations of each inhibitor for 30 min prior to stimulation with DA.

### MTT assay

3-(4,5-Dimethylthiazol-zyl)-2,5-diphenyltetrazolium bromide (MTT) assay was used to evaluate the effect of DA on cell viability and to determine the non-cytotoxic concentrations. Briefly, HCT-116 cells (10^5^) were seeded on 96 well plates and incubated with DA at various concentrations (0, 1, 20, 40, 80 and 100 μM) for 48 h. Thereafter, the medium was changed and MTT (1 mg/ml) was added to each well and incubated in the CO_2_ incubator for 4 h. The viable cell number was directly proportional to the production of formazan crystals which, following adding isopropanol 100 μl to dissolve and the absorbance of each well was obtained using a Bio-Rad Microplate reader at wavelength of 490 nm.

### Semi-quantitative RT-PCR

Semi-quantitative RT-PCR was performed as described previously [15]. Total RNA was isolated from cells using Trizol (BBI, Kitchener, Ont., Canada), and samples of total RNA were quantified by reading optical density at 260 nm. mRNA was reverse transcribed using a first strand cDNA synthesis kit (Fermentas, Glenn Burnie, Md., USA) according to the manufacturer’s instructions. One microgram of cDNA was amplified in the standard reaction mixture using 2× PCR MasterMix (TIANGEN, China) and the forward and reverse primers for hBD-2 and β-actin, an internal standard. The forward and reverse primers and product sizes were as follows: hBD-2 (255 bp) forward: 5′-CCAGCCATCAGCCATGAGGGT- 3′; reverse: 5′-GGAGCCCTTTCTGAATCCGCA- 3′. For β-actin (300 bp), forward: 5′-TCACCCACACTGTGCCCATCTACGA- 3′; reverse: 5′-CAGCGGAACCGCTCATTGCCAATGG- 3′. Amplification proceeded in a PCR Thermal Cycler (Takara PCR Thermal Cycler MP; Takara Bio, Otsu-shi, Japan) using 32 cycles consisting of 94 °C for 60 s, 62 °C for 30 s, and 72 °C for 120 s. The PCR products were verified by electrophoresis in a 1.2 % agarose gel and detected by ethidium bromide staining. Detectable fluorescent bands were visualized with an ultraviolet transill. The values of densitometry of the mRNA bands were acquired using ImageJ software (National Institutes of Health, USA) and then normalized with β-actin.

### Western blot analysis

After cells were treated with DA or inhibitors, total cell extracts were prepared in ice-cold lysis buffer (150 mmol/L NaCl, 1 mmol/L phenylmethylsulfonyl fluoride, 1 mg/mL aprotinin, 1 mg/mL pepstatin, 1 mmol/L pervanadate,1 mmol/L EDTA, 1 % igepal, 0.25 % deoxycholic acid,1 mmol/L NaF, and 50 mmol/L Tris–HCl (pH 7.4)). The lysates were centrifuged at 12 000 r/min for 10 min, and the resulting supernatants were assessed with a Bio-Rad assay kit using bovine serum albumin as the standard. Equal amounts of protein (40 μg/lane) were separated by SDS PAGE and blotted onto 0.22 μm polyvinylidene difluoride membranes (Bio-Rad Laboratories, Hercules, Calif., USA). The membranes were blocked in TBST (Tris-buffered solution, pH 7.6, 0.05 % Tween 20) containing 5 % nonfat dried milk. Membranes were probed overnight at 4 °C with specific primary antibodies including anti-hBD-2, anti-p-p38 (Thr180/182), p38 and anti-β-actin (Santa Cruz, Calif., USA); anti-ERK1/2, anti-p-ERK1/2, anti-JNK, anti-p-JNK (Cell Signaling Technology Inc, USA) . After 3 washes, the blots were subsequently incubated with appropriate secondary antibodies with horseradish peroxidase (HRP, ZSGB-BIO) at room temperature for 1 h and developed in the ECL Western blot detection reagents (Beyotime, Jiangsu, China). The values of densitometry of the protein bands were acquired using ImageJ software (National Institutes of Health, USA) and then normalized with β-actin.

### Statistics

Results were presented as mean ± SD. Data were statistically evaluated using one-way analysis of variance (ANOVA) followed by Dunnett’s test by SPSS 13.0 software (SPSS, Chicago, USA). A *P* value < 0.05 was considered as statistically significant.

## Results

### Cytotoxicity of DA on HCT-116 intestinal epithelial cells

**Fig. 2:**
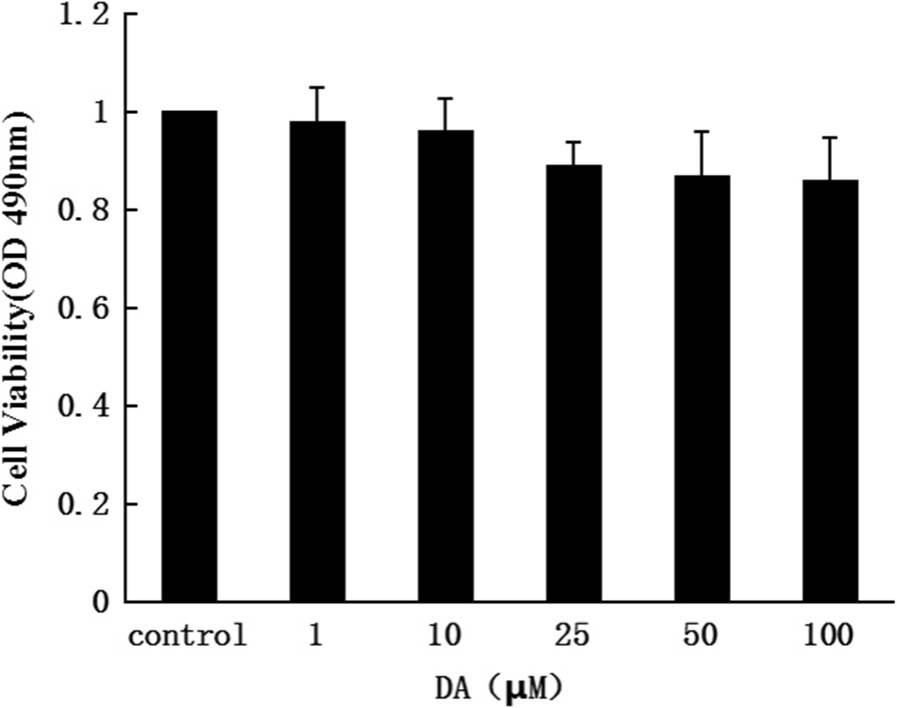
Effects of DA on HCT-116 intestinal epithelial cell viability. HCT-116 intestinal epithelial cells were treated with various concentrations (1, 10, 25, 50, 100 μM) of DA for 48 h. Cell viability was analyzed by MTT assay. DMSO served as the vehicle control. Data were shown as mean ± SD by six parallel experiments. Statistical analysis was performed with one-way ANOVA and Dunnett’s test

First, we determined the cytotoxicity of DA by treating HCT-116 intestinal epithelial cells (10^5^ cells) with DA at various concentrations (0–100 μM) for 48 h followed by MTT assay. In comparison with that of vehicle control (DMSO), the viability of HCT-116 cells was a little decreased with increased DA. The viability of 100 μM group was only 14 % less than control group, and the difference between DA treatment groups and control group had no statistical significance (Fig. 2). Therefore 0–100 μM was applied in all subsequent experiments.

### Expression of hBD-2 mRNA

**Fig. 3:**
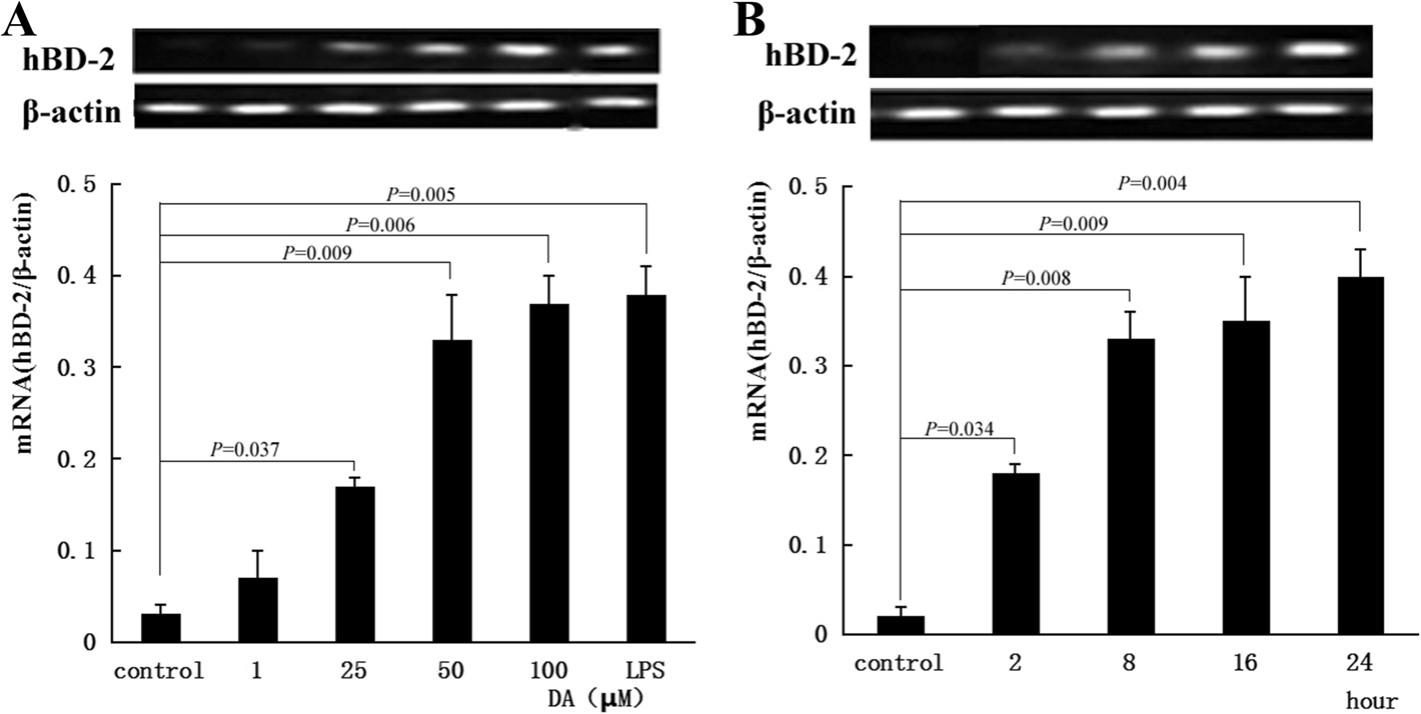
Effects of DA on HCT-116 intestinal epithelial cells hBD-2 mRNA expression. Intestinal epithelial HCT-116 cells were treated with or without different concentrations of DA (1, 25, 50, 100 μM) for 16 h (**a**), or DA 100 μM for different time (**b**). DMSO was used as the vehicle control. LPS (5 μg/ml) was used as the positive control. The total RNA was extracted from cells and analyzed for *hBD-2* or *β-actin*, an internal control by RT-PCR. The results of the RT-PCR were depicted as mean ± SD of three independent experiments. Statistical analysis was performed with one-way ANOVA and Dunnett’s test

In this study, we used different concentrations of DA (0, 1, 25, 50 and 100 μM) to stimulate the HCT-116 intestinal epithelial cells for 16 h and 100 μM DA for different time intervals (0, 2, 8, 16 and 24 h), the results revealed that the DA can up-regulate the expression of the hBD-2 mRNA. The optimal concentration 100 μM, the maximal expression of hBD-2 mRNA occurred after 24 h (Fig. 3). There was a dose-dependent and time-dependent enhancement expression of hBD-2 mRNA upon treatment with DA. It was important to note that expression of the housekeeping gene β -actin was not affected by DA.

### Expression of hBD-2 protein

**Fig. 4:**
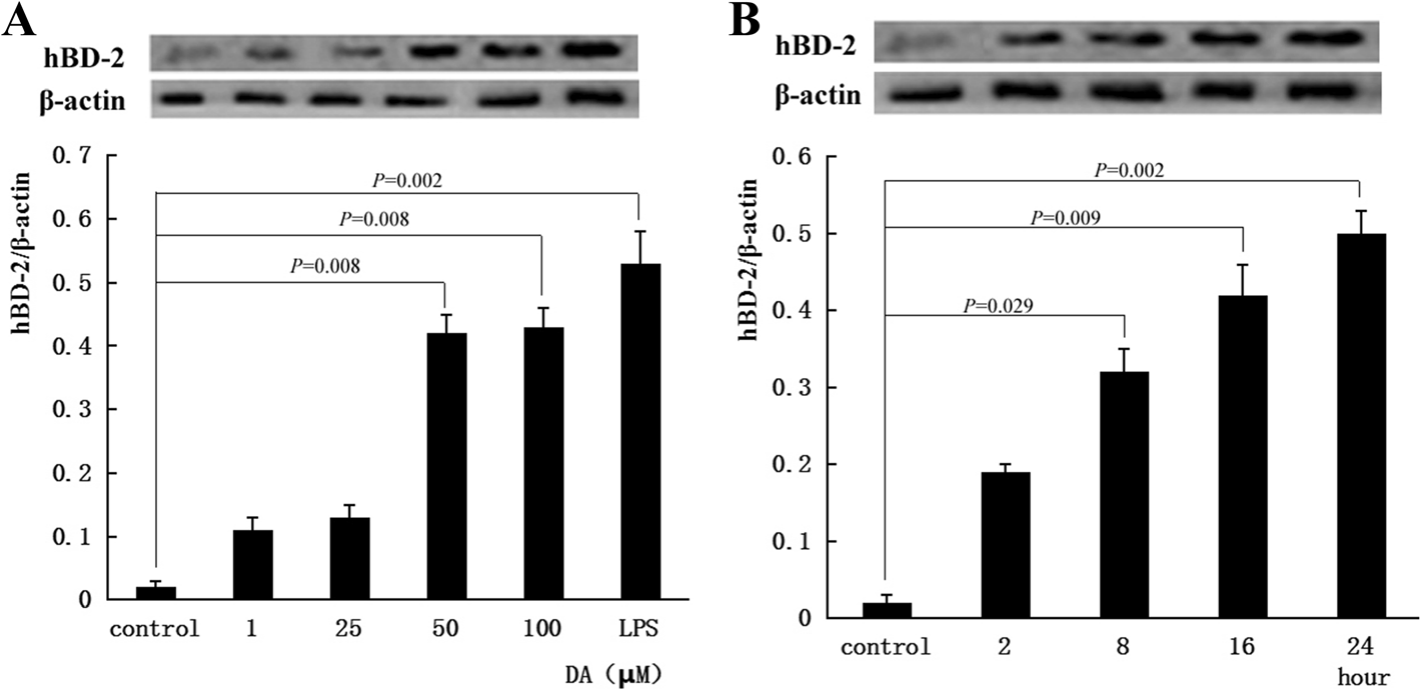
Effects of DA on HCT-116 intestinal epithelial cells hBD-2 protein expression. HCT-116 intestinal epithelial cells were treated with or without different concentration of DA (1, 25, 50, 100 μM) for 16 h (**a**), or DA 100 μM for different time (**b**). DMSO was used as the vehicle control. LPS (5 μg/ml) was used as the positive control. Whole cell lysates were prepared and used for western blot with anti-hBD-2 antibody. β-actin served as an internal control. The values of densitometry of the protein bands were acquired using ImageJ software (National Institutes of Health) and then normalized with β-actin and expressed as relative signal intensity. All values were expressed as mean ± SD of three independent experiments. Statistical analysis was performed with one-way ANOVA and Dunnett’s test

As revealed in Fig. 4, hBD-2 protein expression was detected by Western blot correlated with the hBD-2 mRNA expression. The expression of hBD-2 protein was significantly enhanced by DA, the optimal concentration was 100 μM and the maximal expression of hBD-2 protein occurred after 24 h.

### Mechanism of hBD-2 expression induced by DA

**Fig. 5:**
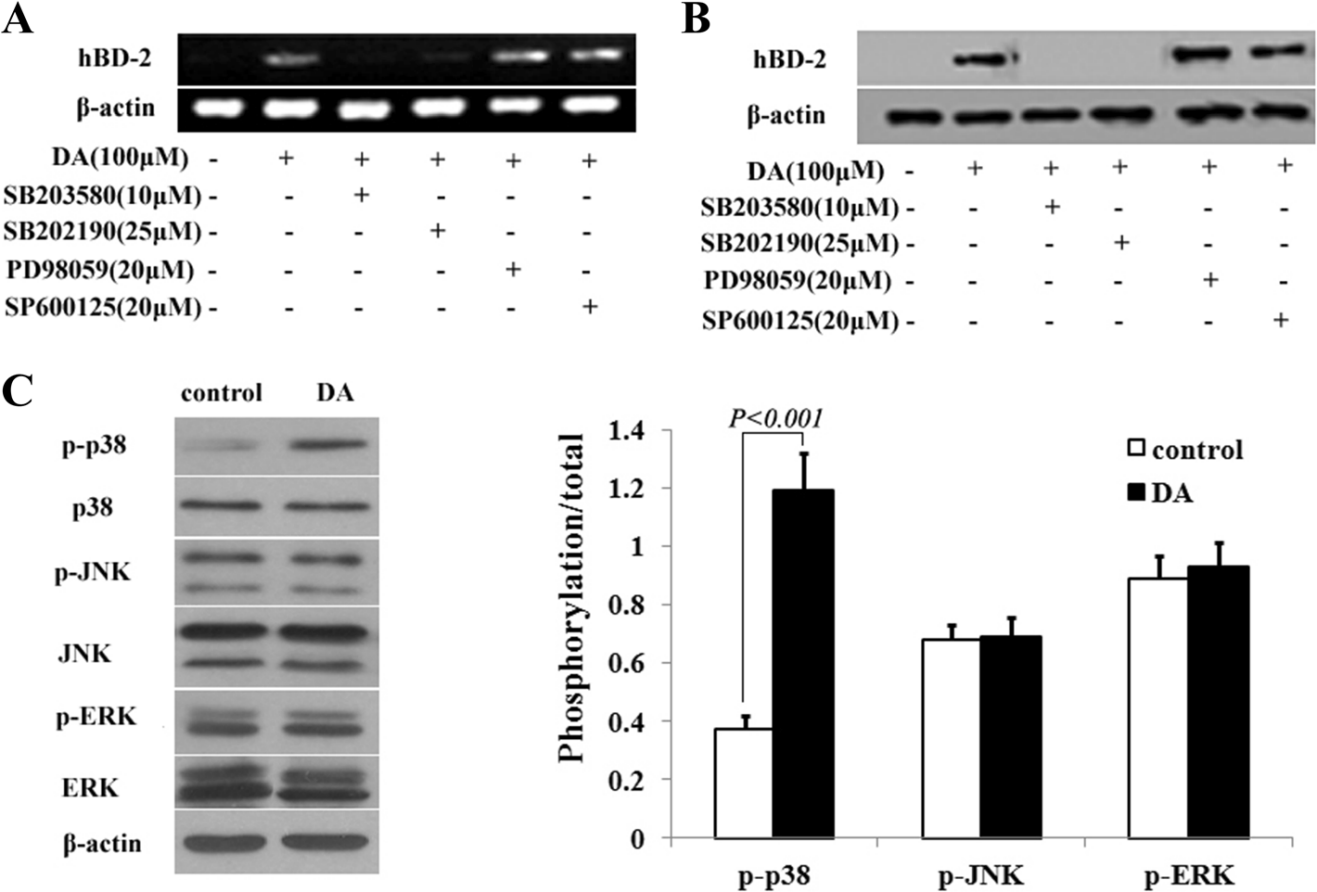
Role of MAPK signaling pathway in DA-induced hBD-2 expression. HCT-116 intestinal epithelial cells were pretreated with indicated concentrations of chemical inhibitors for 30 min, and then treated with 100 μM DA for 16 h, *β-actin* served as an internal control. Total RNA was extracted from cells and analyzed for *hBD-2* by RT-PCR (**a**). In addition, whole cell lysates were prepared and used for western blot with antibodies *hBD-2* (**b**). HCT-116 intestinal epithelial cells were treated with 100 μM DA for 30 min, cell lysates were harvested for the detection of phospho-p38 MARP, ERK, and JNK by western blot, *β-actin* served as an internal control. ‘p’ indicates the phosphorylated form of the protein. The values were expressed as mean ± SD of three independent experiments (**c**)

The previous results showed DA could induce the expression of hBD-2 mRNA and protein in a dose- and time-dependent manner. So we further investigate the possible mechanism of hBD-2 up-regulating in HCT-116 intestinal epithelial cells. Both mRNA and protein expression in responsive to DA was attenuated by pretreatment with two p38 inhibitors (SB203580 and SB202190) respectively, but not by ERK inhibitor PD98059, or JNK inhibitor SP600125. Moreover, we found the phospho-p38 but not phospho-ERK and phospho-JNK was increased in DA treated HCT-116 intestinal epithelial than control (Fig. 5).

## Discussion

Both DA and Androg are main active components in *A. paniculata* which are popular used for all kinds of infection and inflammation in Asia. They have the similar structure and both are labdane diterpenoids. The only difference is the dehydroxylation in C-14 for DA comparing to Androg (Fig. 1). Consequently, they have the similar actions in many conditions [18, 19]. We have found that both Androg and DA could enhance innate immunity by stimulate hBD-1 and hBD-2 expression in respiratory tract epithelial cells [20]. However, it has remained unclear whether they have the same effect in intestinal epithelial cells. Therefore, we first added different concentrations of Androg and DA into culture HCT-116 cells, found DA had less cytotoxic effect for HCT-116 cells than Androg (IC_50_ DA >200 μM vs IC_50_ Androg 10.67 μM for HCT-116 cells 48 h). As the lower toxicity of DA, DA succinate (trade name: Yan-Hu-Ning Injection)) is more popular used for injection in clinical for the treatment of viral pneumonia and upper respiratory tract infections in China [21, 22]. Therefore we focused on observing the effect of DA on hBD-2 expression in HCT-116 cells in this study. We found the level of mRNA and protein of hBD-2 increased 11 and 20 times comparing with control respectively in DA treatment group at non-toxic concentration 100 μM. The effect of DA induction hBD-2 expression was maintained for 24 h, which longer than human lung epithelial cells expression hBD-1.

hBD-2 plays important roles in innate defence system at gastrointestinal (GI) tract mucosal surfaces. Unlike hBD-1 constitutively expression within GI tract and mostly remain stable during inflammation and infection, hBD-2 is induced upon pathogen [23–25]. hBD-2 is seldom expressed in normal colon but its expression is significantly increased in inflamed colonic epithelium [26] . It has been shown that certain probiotic strains can induce the secretion of antimicrobial peptides (such as hBD-2) by intestinal cells and probiotics have already been used as “ecological treatments” in gastrointestinal infections such as diarrhoea, dysentery [27]. At the same time, scientists pay attention to looking for defensins inducer from Chinese herbal medicines such as *Astragalus membranaceus (Fisch.) Bunge., Coptis chinensis Franch, Houttuynia cordata Thunb., Isatis tinctoria* and Lentinan, which will also appear as attractive strategies for these diseases. From our results, DA was proved to be a defensins inducer and would be beneficial for the rehabilitation of intestinal infection.

For the promoter region of the hBD-2 gene contains binding sites for AP-1, and AP-1 is a transcription factor that can be activated by different MAPK pathways, including the p38, ERK and JNK pathway [28], we investigated whether MAPK signalling pathways were involved in DA induced hBD-2 expression in human intestinal epithelial cells. The results showed that it was the specific inhibitors of p38 MAPK but not the inhibitors of ERK and JNK significantly attenuated DA-induced hBD-2 gene expression. When measured the p-p38 (p38 active form) we found the p-p38 increased in DA treated HCT-116 cells, however the p-ERK (ERK active form) and p-JNK (JNK active form) had no significant change. Our findings on the signal passway of DA induced hBD-2 expression in human intestinal epithelial cells are partial consistent with that of a previous study by Gan et al. [12], which reported that paeoniflorin, a substance came from herbal plant peony upregulates β-defensin-2 expression in human bronchial epithelial cell through the p38 MAPK, ERK, and NF-κB signaling pathways. Several studies revealed different inducer may induce hBD-2 expression through different signalling pathways in different cells. *Bacteroides fragilis* enterotoxin induces hBD-2 expression in intestinal epithelial cells via p38/NF-κB-dependent pathway [29]. *Escherichia coli* Nissle 1917 induces hBD-2 expression in intestinal epithelial cells via NF-κB and AP-1 signalling pathways [30]. Avocado sugar modulates the hBD-2 expression through toll-like receptor-2 and ERK/MAPK activation in human keratinocytes [13]. As DA is a potential NF-κB inhibitor [31, 32], whether NF-κB is involved in hBD-2 expression in intestinal epithelial cells need to further confirm. It is still a question whether DA will lead to more serious inflammatory for upregulation hBD-2. Especially for those associated with a high level of hBD-2 gut inflammation such as Crohn’s disease [33, 34]. Immune mediators often have dual-direction regulation like the water extract of *Houttuynia cordata* Thunb [35] and paeoniflorin,which could upregulate β-defensin-2 expression in human bronchial epithelial cell at the same time downregulated β-defensin-2 expression in mice colonic mucosa with oxazolone-induced colitis [36]. The existing evidences have described DA is a immune mediator. DA could significantly reduce the level of IL-1β, IL-6 and TNF-α in the bronchoalveolar lavage fluid of the LPS-induced acute lung injury mouse [37]. DA could dose-dependently inhibited ovalbumin-induced increases in total and eosinophil counts, IL-4, IL-5, and IL-13 levels in lavage fluid in a mouse asthma model [31]. Therefore, DA may not enhance inflammatory at the same time of hBD-2 inducing in GI tract.

## Conclusions

In summary, we have demonstrated that exposure of intestinal epithelial cells to DA caused the activation of a signaling cascade involving p38 MAPK up-regulation expression of hBD-2 in intestinal epithelial cells. This is one main reason why DA could be used for intestinal infection.

## Abbreviations

DA: Dehydroandrographolide
hBD-2: human β –defensin-2
RT-PCR: Reverse transcription polymerase chain reaction
MAPK: Mitogen-activated protein kinase
ERK: Extracellular regulated protein kinases
JNK: c-Jun N-terminal kinase
DMSO: Dimethylsulphoxide
NF-κB: Nuclear factor kappa-light-chain-enhancer of activated B cells
